# Efficacy of Transgenic Maize LD05 Against Fall Armyworm (*Spodoptera frugiperda*)

**DOI:** 10.3390/plants14162504

**Published:** 2025-08-12

**Authors:** Wenlan Li, Xiang Gao, Xinwei Hou, Zhaohua Ding, Zhaodong Meng, Runqing Yue

**Affiliations:** 1 Maize Research Institute, Shandong Academy of Agricultural Sciences, Shandong Key Laboratory of Maize Biological Breeding, National Engineering Center of Wheat and Maize, Jinan 250100, China; liwenlantutu@126.com (W.L.); houxinwei92@163.com (X.H.); dingzhaohua76@163.com (Z.D.); mengzhd981@126.com (Z.M.); 2 JoinHope Seeds Co., Ltd., Changji 831100, China; gaoxiang1980jsh@163.com

**Keywords:** agronomic traits, efficacy, expression, fall armyworm, transgenic maize LD05

## Abstract

The fall armyworm (*Spodoptera frugiperda* (J.E. Smith)), which invaded China in 2018, has caused severe corn yield losses and increased pesticide application frequency. *Bacillus thuringiensis* (Bt)-based genetically modified corn represents an environmentally friendly and effective strategy for managing *S. frugiperda*. The transgenic corn LD05 harbors the *m2cryAb-vip3A* insect-resistant fusion gene, which has demonstrated potent inhibitory effects against fall armyworm and is currently in the phase of applying for safety certification. Here, we evaluated the inhibitory efficacy of LD05 against *S. frugiperda* through laboratory and field experiments during 2022–2024. The LC_50_ and LC_95_ of M2CryAb-VIP3A against fall armyworm were 0.024 μg/cm^2^ and 0.508 μg/cm^2^, respectively; and the GLC_50_ and GLC_95_ were 0.142 μg cm^−2^ and 0.556 μg cm^−2^, respectively. M2CryAb-VIP3A expression of LD05 varied significantly across tissues, and remained stable between generations. Bioassays revealed significant tissue-specific differences in fall armyworm larval mortality for LD05 corn tissues, ranked as V5-stage leaves > R3-stage kernels > R1-stage silks. Field trials demonstrated that LD05 corn significantly reduced fall armyworm larval populations, leaf damage incidence, and plant damage incidence compared to non-Bt control Zheng58. Agronomic trait analysis showed no significant differences between LD05 and Zheng58. These results indicate that LD05 has a significant inhibitory effect on fall armyworm, which is an effective strategy for the comprehensive management of fall armyworm in China.

## 1. Introduction

Maize (*Zea mays*), China’s primary grain crop, is critical to national food security. In December 2018, the invasive lepidopteran pest fall armyworm ((*Spodoptera frugiperda* (J.E. Smith) Lepidoptera: Noctuidae, FAW) was first detected in Yunnan Province, having migrated from Myanmar [1,2]. The FAW has a strong carnivorous nature, a strong reproductive capacity, and an extremely strong migratory ability. It can fly hundreds of kilometers in one night, which is an important reason for its global outbreak [3,4,5]. By 2019, FAW had infested >20 provinces across China, affecting 1.065 million hectares of cropland [6,7], emerging as a major threat to maize production. FAW larvae inflict damage on maize tissues—leaves, stems, and ears—through diverse feeding behaviors that intensify with larval development. This pest causes annual economic losses estimated at USD 5.4–47 billion to China’s maize industry [8]. Consequently, the Chinese Government has implemented integrated pest management (IPM) strategies to mitigate FAW-driven losses.

Chemical control is the primary FAW management strategy. However, due to the long-term and high-dose use of pesticides [9], extensive insecticide use has caused widespread FAW resistance—escalating from 1980s organophosphates (e.g., trichlorfon) [10] to 1990s pyrethroids (e.g., cypermethrin) [11], reaching medium-high levels. Only newer insecticides (emamectin benzoate, chlorantraniliprole) maintain low resistance and efficacy [12]. In China, the resistance level of the invading fall armyworm to new pesticides such as emamectin is relatively low, but its resistance to traditional insecticides is at a medium to high level [13]. Such approaches conflict with sustainable agriculture, necessitating alternative resistance-mitigation strategies.

Insect-resistant genetically modified (GM) corn plays a significant role in increasing production, ensuring food safety, protecting the environment and promoting economic development. Global FAW management increasingly relies on GM maize [14,15]. Since its 1996 U.S. debut, GM maize cultivation reached 21.1 million hectares (317 million mu) by 2019 [16]. This approach effectively suppresses FAW while significantly reducing insecticide usage [17].

To leverage genetically modified insect-resistant maize for FAW control and pesticide reduction, China has approved multiple transformation events (e.g., Ruifeng125, DBN3601T) and initiated FAW sensitivity studies. The *m2cryAb-vip3A* gene is a novel insect-resistant fusion gene designed through targeted modification and optimization, and it holds independent intellectual property rights [18]. The protein expressed by it has the activity of both Cry1Ab and Vip3A proteins, which can effectively expand the insecticidal spectrum. The *m2cryAb-vip3A* and herbicide-tolerant gene *bar* were introduced in series into the HiII recipient corn to obtain a transformation event with clear molecular characteristics [19]. The target gene was a single copy insertion, and no other extraneous sequences were inserted [18]. Using Zheng58 as the reincarnation parent, after 8 consecutive hybridizations and 2 self-pollinations, a new type of insect-resistant and herbicide-tolerant transgenic corn LD05 was obtained and has now entered the stage of safety certificate approval. The transgenic corn LD05 exhibits high resistance to key lepidopteran pests (*Ostrinia furnacalis* (Guenée), *Mythimna separata* (Walker), *Helicoverpa armigera* (Hübner), and *Spodoptera frugiperda* (J. E. Smith)*)* [19]. However, no systematic assessment exists regarding FAW sensitivity to M2CryAb-VIP3A in LD05 across southern China, including Hainan Province. This study bridges this gap through laboratory bioassays and field trials evaluating FAW sensitivity to M2CryAb-VIP3A and LD05’s efficacy against FAW infestation. The results show that the fusion protein expressed by LD05 has dose-dependent toxicity to FAW, and the expression level is sufficient to provide field protection, which provides an important theoretical and technical basis for the promotion of insect-resistant GM corn (such as LD05) in southern China’s agricultural systems.

## 2. Materials and Methods

### 2.1. Plant Materials

The recipient conventional maize line for transgenic *m2cryAb-vip3A* insect-resistant maize LD05 was Zheng58. Both Bt maize LD05 and non-Bt maize Zheng58 used in laboratory and field experiments were provided by the Shandong Academy of Agricultural Sciences. The plants for the expression and laboratory bioassay studies were grown in field of Hainan over three consecutive growing seasons (2022–2024), and no insecticides were used throughout the entire growth period, and other management was the same as conventional field management. The tissues used in ELISA analysis included leaves, roots and stems from the seedling stage (V5 stage), elongation stage (V10 stage) and tasseling stage (R1 stage), pollen and filaments from the silk stage (R1 stage), and leaves, roots, stems, bracts. Samples were taken from every three plants as a biological repetition, and grains from the milk stage (R2 stage) and mature stage (R3 stage). Each 3 plants was a duplicate, and the samples were quick-frozen in liquid nitrogen and stored at −80 °C for later use.

### 2.2. Bioassay of FAW Susceptibilities to M2CryAb-VIP3A Proteins

The FAW population was provided by Henan Jiyuan Baiyun Co., Ltd. (Jiyuan, China). This population was collected from Guangdong, China in 2020. This population had been laboratory-reared for >2 generations with consistent genetic background, and had no prior exposure to chemical pesticides or insect-resistant transgenic plants in the past two generations. The proteins used for bioassay were expressed in prokaryotes, purified and then subjected to gradient dilution. The work of the equivalence of the prokaryotic-produced and plant-produced protein has been conducted and the biological equivalence has been established (Li, unpublished data).

Bioassays employed the surface coating method: First, approximately 1 mL liquid artificial diet (~1 g) was dispensed into 24-well plates and gently agitated to cover the bottom uniformly. After diet solidification, 20 μL M2CryAb-VIP3A protein solutions at five concentrations (0.08, 0.16, 0.4, 0.8, 1.6 μg/cm^2^) were applied to each well, with PBS as control. Plates were gently shaken to distribute solutions evenly over diet surfaces, then air-dried in a fume hood. Twenty-four neonate larvae (2–12 h post-hatching) were inoculated per concentration, with three replicates. Plates were maintained at 25 ± 2 °C under 40–60% RH and 14:10 (L:D) h photoperiod.

Mortality was assessed after 5 days. Larvae unresponsive to caudal brush stimulation or failing to reach second instar were recorded as dead. Calculations:

- Mortality (%) = (Dead larvae/Total larvae) × 100
- Corrected mortality (%) = [(Treatment mortality − Control mortality)/(1 − Control mortality)] × 100
- Weight inhibition (%) = [(Control weight gain − Treatment weight gain)/Control weight gain] × 100

### 2.3. Enzyme-Linked Immunosorbent Assay (ELISA)

M2cryAb-vip3A protein levels were quantified using a commercial ELISA kit (Wuhan Lauken Biotechnology Co., Ltd. (Wuhan, China)) [20]. After 30 min equilibration at room temperature, all reagents were vortexed prior to use. Standards were diluted and samples prepared according to manufacturer protocol. Following sequential steps of sample/standard addition, room-temperature incubation in darkness, plate washing, enzyme conjugate addition, repeated washing, and color development [20], absorbance was measured at dual wavelengths (450/630 nm) using a microplate reader.

### 2.4. Bioassays Using Plant Tissues

The FAW population source and rearing conditions matched Section 2.2.

Resistance of transgenic maize LD05 was evaluated by feeding FAW neonate larvae (<24 h old) with young leaves (V5 stage), unpollinated silks (R1 stage), and kernels (R3 stage). Methods: Leaves/Silks: twenty random plants were sampled. Tissues were placed in rearing containers; each sample was infested with five neonate larvae (20 replicates). Kernels: five kernels from each of the 20 plants were placed in multi-well rearing boards (one kernel per well), each infested with one larva (20 replicates). Fresh tissues were replenished daily based on consumption. Survival was recorded every day for a total of 5 days; corrected mortality was calculated as in Section 2.2.

### 2.5. Field Trials

**Figure 1:**
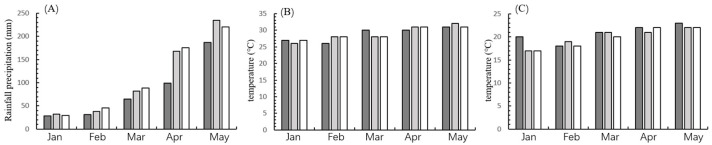
Rainfall precipitation and temperature of experimental location from 2022 to 2024. (**A**) Monthly rainfall precipitation, (**B**) Monthly average temperature, (**C**) Monthly mean low temperature. The dark grey columns represent 2022, the light grey columns represent 2023, and the white columns represent 2024.

Field experiments were conducted from January to May in 2022, 2023, and 2024 at Plot E1 of the Phase II Biological Breeding Zone (18°26′16″ N, 109°12′12″ E). Each trial utilized a randomized block design with three replicates per treatment, where each plot measured 100 m^2^ (5 m × 20 m) and was separated by 1.0 m isolation paths. Plants were spaced at 60 cm row intervals and 32 cm within rows. Field management followed standard agricultural practices, with no pesticides applied to allow natural *Spodoptera frugiperda* (FAW) infestation. Monthly precipitation, mean temperature, and minimum temperature data for Yazhou District, Sanya City, during 2022–2024 were obtained from the Hainan Meteorological Bureau (http://hi.cma.gov.cn/) and are shown in Figure 1.

Field metrics included larval density (the number of FAW on the entire plant), leaf damage scores (based on a severity score suggested by CIMMYT [21]), and plant damage incidence (checking the whole corn plant, especially leaf and whorl at the V stage, silk, and kernels on the ear tip at the R stage), evaluated via a W-shaped five-point sampling method (20 plants per point) at the V5, R1, and R3 growth stages [22].

Specific focus was placed on V5-stage leaves, R1-stage silks/leaves/stems, and R3-stage kernels/leaves/stems/cobs. The control efficacy of transgenic corn LD05 against FAW larvae at each stage was calculated as Efficacy % = (Number of larvae of non-transgenic plants − Number of larvae of transgenic plants)/Number of larvae of non-transgenic plants × 100.

### 2.6. The Agronomic Traits Detection

To evaluate whether exogenous gene insertion impacts maize agronomic traits, transgenic corn LD05 and the control line Zheng58 were comparatively evaluated for three consecutive years (2022–2024) across key agronomic parameters, including growth stages (tasseling stage, silking stage, anthesis stage, and growth duration), plant height, ear height, and ear-related traits (ear row number, cob diameter, 100-kernel weight, and pollen grain diameter).

### 2.7. Pollen Viability Detection

Three transgenic corn LD05 plants and three non-transgenic control Zheng58 plants were selected and set as three biological replicates. The pollen of LD05 and Zheng58 were collected and scattered evenly in the pollen germination medium (the medium formula is 10% sucrose, 0.0005% boric acid, 10 mM CaCl_2_, 0.05 mM KH_2_PO_4_, 6% PEG4000, 0.3% Noble Agar). The diameter of the pollen grains was measured immediately through a microscope, and 30 pollen grain diameters for each plant were counted. Then, in an environment with a humidity of 70–85%, the pollen germination in each petri dish was observed after being placed for different periods of time (1 h, 3 h, 6 h) at conditions of 25 °C, 30 °C, and 35 °C, respectively. Three dishes of pollen were cultured in each treatment group, and the number of pollen observed in each dish was not less than 200. If the length of the pollen tube is greater than the diameter of the pollen grain, it is regarded as germinating pollen.

### 2.8. Statistical Analyses

The toxicity and growth inhibition efficiency of Bt protein against *S. frugiperda* larvae were estimated using probit analysis. Expression levels of the M2CryAb-VIP3A protein across different tissues of transgenic maize LD05 and corrected mortality rates of *S. frugiperda* (FAW) larvae were analyzed using Tukey HSD test. Field trial data were analyzed using Tukey HSD test and two-sample t-test to assess significant differences in live FAW counts, leaf damage scores, and plant damage incidence rates between transgenic LD05 and non-transgenic Zheng58 at each growth stage. Tukey HSD test analysis was used to compare the significance of differences in the agronomic characters and pollen germination rates. All statistical analyses were performed using SPSS 13.0.

## 3. Results

### 3.1. Sensitivity of Spodoptera frugiperda Larvae to M2CryAb-VIP3A

The insecticidal activity of M2CryAb-VIP3A protein against fall armyworm (FAW) was evaluated through bioassays (Table 1). The protein demonstrated potent toxicity, with LC_50_ and LC_95_ values of 0.024 μg cm^−2^ and 0.508 μg cm^−2^, respectively. Additionally, M2CryAb-VIP3A significantly inhibited larval growth, exhibiting growth inhibition concentration (GIC) values of 0.142 μg cm^−2^ (GIC_50_) and 0.556 μg cm^−2^ (GIC_95_).

### 3.2. Expression Analysis of M2CryAb-VIP3A Protein in Transgenic Maize LD05

**Figure 2:**
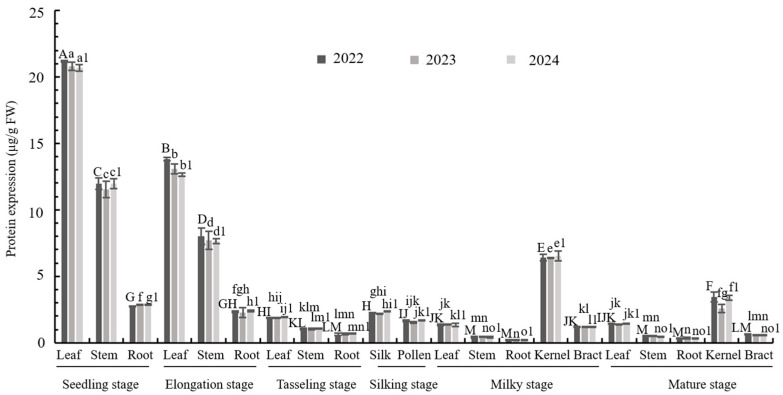
Tissue-specific expression of M2CryAb-VIP3A protein in transgenic maize LD05. Error bars represent standard error (SE) of the mean. Different uppercase letters or lowercase letters and numbers above bars indicate statistically significant differences (*p* < 0.05) among tissues of different developmental stages, as determined by Tukey HSD test. Shared letters indicate no significant difference.

The results demonstrated a consistent and stable expression of M2CryAb-VIP3A across all three annual batches, with comparable expression patterns observed each year (2022, *F*_20,42_ = 3852.102, *p* = 0; 2023, *F*_20,42_ = 1951.848, *p* = 0; 2024, *F*_20,42_ = 4011.269, *p* = 0). The protein concentration exhibited significant tissue-specific variation, ranging from 0.21 to 21.15 μg/g fresh weight (FW) (Figure 2). Notably, the highest accumulation occurred in seedling-stage leaves (20.67–21.15 μg/g FW), with a progressive decline in expression as leaves matured. The lowest expression levels were detected in roots at the milk maturity stage (0.21–0.24 μg/g FW). Interestingly, mature kernels showed reduced M2CryAb-VIP3A content compared to milk-stage kernels. As expected, no M2CryAb-VIP3A expression was detected in any tissues of the non-transgenic control Zheng58.

### 3.3. Bioassay of Transgenic Maize LD05 Tissues Against FAW Neonates

**Figure 3:**
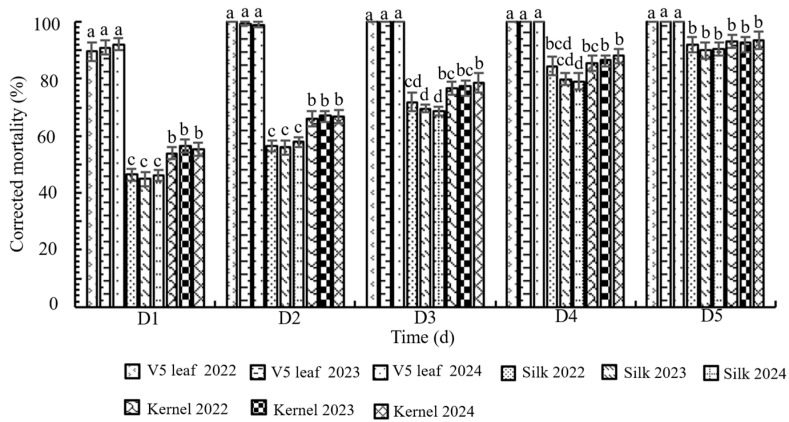
Corrected mortality of FAW neonates that fed on different tissues of transgenic maize LD05 for different durations. Error bars indicate standard error (SE) of the mean. Different lowercase letters above bars denote statistically significant differences (*p* < 0.05) in mortality rates among tissues as determined by Tukey HSD test. Shared letters indicate no significant difference. D represents the number of days after inoculation.

The bioassay results revealed consistent mortality patterns across years for each tissue type, while showing significant variation among different tissues (Figure 3). One-way ANOVA confirmed no significant interannual variation in corrected mortality for any given tissue, but detected highly significant differences among tissue types at all evaluation time points (day 1, *F*_8,18_ = 227.595, *p* = 0; day 2, *F*_8,18_ = 351.080, *p* = 0; day 3, *F*_8,18_ = 136.563, *p* = 0; day 4, *F*_8,18_ = 50.631, *p* = 0; day 5, *F*_8,18_ = 11.363, *p* = 0) (Figure 3). Consistent with the expression profile of M2CryAb-VIP3A, V5-stage leaves demonstrated the highest insecticidal activity, inducing >80% corrected mortality within 24 h and reaching 100% lethality by 72 h post-exposure. This corrected mortality rate was significantly higher (*p* < 0.05) than that observed for R1-stage silks or R3-stage kernels (Figure 3). The overall efficacy ranking of tissues was V5 leaves > R3 kernels > R1 silks, mirroring the relative expression levels of the insecticidal protein.

### 3.4. Field Evaluation of Transgenic Maize LD05 Against FAW During 2022–2024

**Figure 4:**
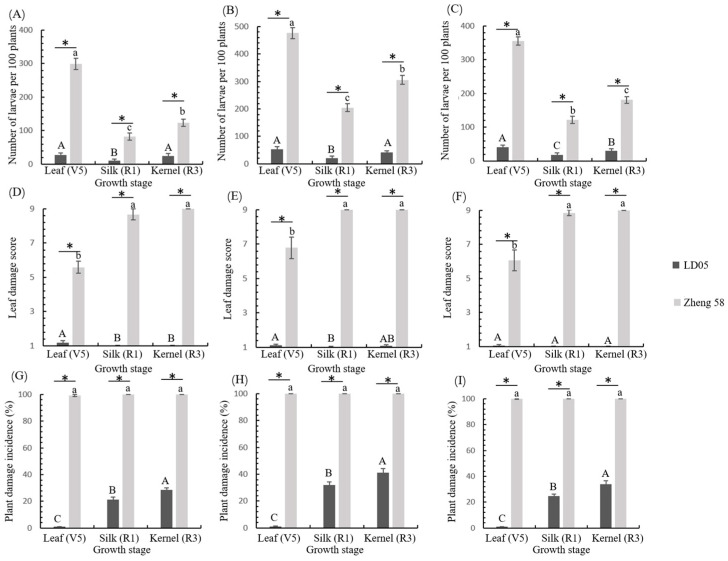
Field trials of transgenic maize LD05 against FAW in different years. (**A**–**C**) FAW larval density dynamics at key developmental stages (V5, R1, R3) in transgenic LD05 versus non-Bt Zheng58: (**A**) 2022, (**B**) 2023, (**C**) 2024. (**D**–**F**) Foliar damage severity (1–9 scale) induced by FAW larvae: (**D**) 2022, (**E**) 2023, (**F**) 2024. (**G**–**I**) Plant damage incidence (% injured plants) across phenological stages: (**G**) 2022, (**H**) 2023, (**I**) 2024. Error bars denote standard error (SE). Asterisks indicate significant differences between genotypes at equivalent growth stages (two-sample *t*-test, *p* < 0.05). Letters above bars denote statistical separation among seasonal measurements within each genotype (Tukey HSD test, *p* < 0.05).

The results of the t-test revealed significantly higher FAW larval populations in non-Bt Zheng58 compared to transgenic LD05 across all three developmental stages (V5, R1, R3) in annual field trials (Figure 4A–C). Interannual variation showed distinct infestation patterns in the conventional cultivar, with peak larval densities recorded in 2023 (V5: 476.2 ± 20.4 larvae/100 plants; R3: 306.0 ± 16.0 larvae/100 plants) (Figure 4B). In contrast, LD05 maintained consistently low pest pressure, with maximum annual larval counts at V5 stage being 26.6 ± 6.2 (2022), 53.0 ± 9.7 (2023), and 41.2 ± 6.4 (2024) per 100 plants (Figure 4A–C). ANOVA indicated that FAW larval populations of non-Bt Zheng58 differed significantly among different developmental stages in 2022 (*F*_2,12_ = 389.345, *p* = 0), 2023 (*F*_2,12_ = 316.191, *p* = 0), and 2024 (*F*_2,12_ = 578.048, *p* = 0), so as Bt maize LD05 (2022, *F*_2,12_ = 11.089, *p* = 0.002; 2023, *F*_2,12_ = 21.222, *p* = 0; 2024, *F*_2,12_ = 16.219, *p* = 0).

Non-Bt Zheng58 exhibited severe leaf damage progression across growth stages, consistently reaching maximum severity scores (Level 9) at reproductive phases (R1 and R3 stages). Transgenic LD05 demonstrated remarkable foliar protection with damage indices never exceeding Level 2 (Figure 4D–F). Two-sample t-tests confirmed significant differences (*p* < 0.05) in leaf damage between genotypes at equivalent developmental stages. ANOVA indicated that leaf damage of non-Bt Zheng58 differed significantly among different developmental stages in 2022 (*F*_2,12_ = 235.031, *p* = 0; 2023), 2023 (*F*_2,12_ = 64.128, *p* = 0), and 2024 (*F*_2,12_ = 106.774, *p* = 0), so as Bt maize LD05 (2022, *F*_2,12_ = 7.929, *p* = 0.006; 2023, *F*_2,12_ = 5.725, *p* = 0.018; 2024, *F*_2,12_ = 4.410, *p* = 0.037).

Complete susceptibility (100% plant damage) was observed in Zheng58 across all trial years and growth stages, while the transgenic line showed substantially reduced injury rates (<50% incidence) with significant differences among different developmental stages in 2022 (*F*_2,12_ = 456.115, *p* = 0), 2023 (*F*_2,12_ = 425.407, *p* = 0), and 2024 (*F*_2,12_ = 450.755, *p* = 0) (Figure 4G–I). Two-sample *t*-tests confirmed significantly higher (*p* < 0.05) plant damage in the conventional cultivar at equivalent phenological stages.

**Figure 5:**
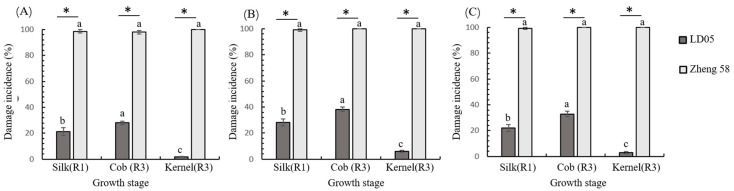
Reproductive tissue susceptibility to FAW infestation in transgenic LD05 and non-Bt Zheng58 across developmental stages. Interannual comparisons of damage incidence in silk (R1 stage), cobs, and kernels (R3 stage): (**A**) 2022, (**B**) 2023, (**C**) 2024. Error bars represent standard error (SE). Asterisks denote significant inter-genotype differences in tissue-specific damage between transgenic LD05 and non-Bt Zheng58 (two-sample *t*-test, *p* < 0.05). Letters indicate intra-genotype variation among reproductive tissues (silk, cob, kernels), as determined by one-way ANOVA with Tukey HSD test (*p* < 0.05).

Further analysis revealed near-complete susceptibility in non-Bt Zheng58’s reproductive tissues, with silk damage (R1 stage), cob injury, and kernel infestation rates all approaching 100% across trial years (Figure 5). Transgenic LD05 exhibited distinct temporal resistance patterns, showing significant stage-dependent damage variation (2022, *F*_2,12_ = 265.270, *p* = 0; 2023, *F*_2,12_ = 425.083, *p* = 0; 2024, *F*_2,12_ = 281.306, *p* = 0) (Figure 5). Notably, the transgenic line maintained <28% silk damage at R1 and <38% cob/kernel damage at R3 stages throughout the study period.

### 3.5. Field Control of Transgenic Maize LD05 Against FAW During 2022–2024

**Figure 6:**
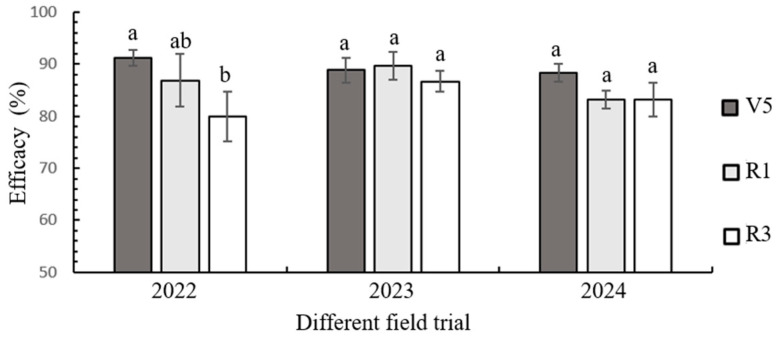
Stage-specific field efficacy of transgenic maize LD05 against FAW larvae in field trials during 2022–2024. Different lowercase letters above bars denote statistically significant differences (*p* < 0.05) in different developmental stages as determined by Tukey HSD test. Shared letters indicate no significant difference.

Multi-year field assessments revealed consistent pest suppression patterns in transgenic LD05 across developmental stages. The cultivar demonstrated sustained efficacy against FAW, with stage-specific control rates showing minimal interannual variation (Figure 6). The results of ANOVA showed that there was no significant difference in the efficacy for each growth stage in each field trial (2023, *F*_2,12_ = 2.164, *p* = 0.158; 2024, *F*_2,12_ = 3.292, *p* = 0.073), except in 2022 (*F*_2,12_ = 9.467, *p* = 0.003). Peak efficacy occurred at the V5 stage (91.19 ± 1.58% in 2022), followed by R1 (89.66 ± 2.64% in 2023), and R3 stages (86.68 ± 2.00% in 2024), establishing a clear efficacy gradient: V5 > R1 > R3 (Figure 6).

### 3.6. Agronomic Traits of Transgenic Maize LD05 During 2022–2024

Under normal planting conditions, no significant differences were observed between transgenic LD05 and Zheng58 in tasseling stage (*F*_5,114_ = 1.809, *p* = 0.117), silking stage (*F*_5,114_ = 1.018, *p* = 0.411), anthesis stage (*F*_5,114_ = 0.800, *p* = 0.552), growth duration (*F*_5,114_ = 1.444, *p* = 0.214), plant height (*F*_5,114_ = 1.336, *p* = 0.254), ear height (*F*_5,114_ = 0.309, *p* = 0.907), ear row number, cob diameter (*F*_5,114_ = 1.506, *p* = 0.913), 100-kernel weight (*F*_5,114_ = 1.324, *p* = 0.259), or pollen grain diameter (*F*_5,174_ = 2.218, *p* = 0.055) (Table 2).

**Figure 7:**
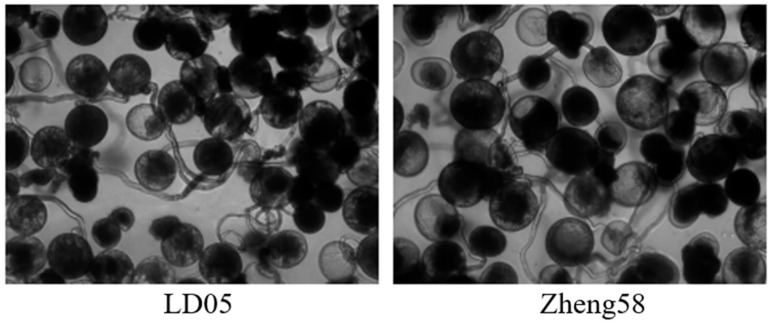
Pollen morphology of transgenic maize LD05 and its non-transgenic Zheng58 at 2 h during in vitro germination.

Additionally, under ambient temperature conditions (no temperature treatment), pollen germination rates of both LD05 and Zheng58 exceeded 75% (Figure 7), confirming high viability of the collected samples. Pollen germination responses of LD05 and Zheng58 to temperature treatments (25 °C, 30 °C, and 35 °C for 1 h, 3 h, and 6 h) are presented in Table 3. Across all treatments, pollen germination rates of both lines exhibited consistent trends: germination declined progressively with extended treatment duration (Table 3). Under identical treatment conditions, no significant differences in pollen germination rates were observed between LD05 and Zheng58 (No Treatment (*F*_5,12_ = 0.626, *p* = 0.683), Treatment at 25 °C for 1 h (*F*_5,12_ = 0.157, *p* = 0.974), Treatment at 25 °C for 3 h (*F*_5,12_ = 0.390, *p* = 0.847), Treatment at 25 °C for 6 h (*F*_5,12_ = 1.361, *p* = 0.305), Treatment at 30 °C for 1 h (*F*_5,12_ = 0.318, *p* = 0.893), Treatment at 30 °C for 3 h (*F*_5,12_ = 0.205, *p* = 0.954), Treatment at 35 °C for 1 h (*F*_5,12_ = 0.684, *p* = 0.644), Treatment at 35 °C for 3 h (*F*_5,12_ = 0.311, *p* = 0.897)). These results indicate comparable pollen activity between the transgenic line and its non-transgenic control.

## 4. Discussion

Since its invasion of China in 2018, fall armyworm (FAW) (*Spodoptera frugiperda*) damage has primarily affected corn-growing regions in southern China [23]. Consequently, field research on FAW has largely been concentrated in southern provinces, such as Yunnan and Sichuan [22,24]. To evaluate the efficacy of transgenic maize LD05 against FAW, we selected Hainan Province for our study, where FAW infestation during the second planting season (January–May) is severe, providing a robust environment for assessing insect resistance.

Previous studies determined the median lethal concentration (LC_50_) of Vip3A protein against FAW from 13 to 1650 ng cm^−2^ [25,26,27,28,29,30,31,32]. The LC_50_ of the M2CryAb-VIP3A fusion protein expressed in LD05 against FAW was significantly lower at 24 ng cm^−2^, implying that M2CryAb-VIP3A was similar to Vip3A in its median lethal concentration to FAW. Furthermore, the innovative M2CryAb-VIP3A fusion protein combines Cry1Ab and Vip3A activities, conferring high resistance against both *Ostrinia furnacalis* (Guenée) and FAW [19]. This dual activity broadens the insect resistance spectrum [19].

M2CryAb-VIP3A protein expression levels in LD05 tissues were significantly different across developmental stages. Consistent with prior findings [24,33], Bt toxin protein expression was highest during early tissue development. The highest expression level of M2CryAb-VIP3A in LD05 was found in V5 stage leaves (19.67–20.15 μg/g FW), and the expression decreased gradually with growth and development. The protein content in the silks is relatively low (2.18–2.35 μg/g FW), which is also consistent with the results of corrected mortality rate of the silks in bioassays tests and the effect of field trials.

Bioassays on transgenic maize DBN3601T (expressing Cry1Ab and Vip3Aa19) indicated that silks were the plant’s most vulnerable tissue to FAW, with a corrected mortality rate of 41.67% [22]. Our results align with this: silks exhibited the lowest corrected mortality against FAW (averaging 90.8% over three years) in LD05, confirming them as the weakest point throughout maize development [22,34]. Conversely, LD05 leaves at the V5 stage demonstrated the highest corrected mortality, followed by kernels. Three consecutive years of tissue-specific bioassays confirmed the stable, generational resistance of LD05 to FAW.

Multi-year field trials (2022–2024) demonstrated significant FAW control by LD05. Larval densities on LD05 were consistently and significantly lower than on non-Bt isoline Zheng58, indicating strong suppression of FAW survival, consistent with other Vip3A-containing events like DBN3601T [33]. Leaf damage scores for LD05 were significantly reduced compared to Zheng58 and consistently remained below level 2 (on a standard scale) over three years, paralleling the efficacy of DBN3601T and Bt11 × MIR162 × GA21 (expressing Cry1Ab and Vip3Aa20) [35,36,37]. Plant damage incidence was also significantly lower in LD05. This consistent efficacy across years may be attributed to relatively stable temperature and rainfall conditions at the experimental site (Figure 1), as environmental stressors like drought can reduce gene expression and compromise insect resistance [19,38].

Field assessments of R1-stage silks and R3-stage cobs/kernels revealed that damage incidence on silks and cobs closely mirrored overall plant damage incidence. This field observation aligns with bioassay results showing silks had the lowest corrected mortality. We hypothesize that this vulnerability facilitates higher FAW invasion rates on silks, potentially leading to increased cob damage by colonizing larvae. The specific mechanisms require further experimental validation. Although LD05 achieved field control efficacy exceeding 80%, it was slightly lower than the weighted efficacy reported for DBN3601T [22]. This discrepancy may stem from FAW’s potential oviposition preference for transgenic corn [22], a factor not accounted for in our control efficacy calculation. Agronomic equivalence between transgenic crops and their controls is a critical consideration. Comparative analysis revealed no significant differences between LD05 and Zheng58 in growth period, plant/ear morphology, or pollen viability. This indicates that the high expression of M2CryAb-VIP3A protein confers effective FAW control without adversely affecting key agronomic traits.

While transgenic crops have significantly reduced pest damage, the expanding cultivation area raises concerns about the accumulation and inheritance of pest resistance [39,40,41,42,43,44]. Although current resistance monitoring shows no significant increase in resistance allele frequency within FAW populations, studies on cotton bollworm (*Helicoverpa armigera*) in Bt cotton regions demonstrate rising tolerance over time [33]. Primary resistance management strategies include the high-dose/refuge strategy and the pyramid strategy. The pyramid strategy involves deploying multiple distinct toxins, either sequentially or concurrently, ideally with different binding sites to minimize cross-resistance risk. Expressing two or more toxins within a single variety is currently considered optimal for delaying resistance, such as DBN3601T [39,40,41,42,43,44]. However, the pyramid strategy mainly integrates multiple genes through hybrid breeding methods, which has some problems such as complex operation and linkage drag. To address technical and industrial resistance management needs, LD05 employs the innovative M2CryAb-VIP3A fusion protein. This single protein combines Cry1Ab and Vip3A activities, expanding the resistance spectrum without generating cross-resistance, thereby enhancing durability.

## 5. Conclusions

This evaluation of the efficacy of transgenic maize LD05 against *S. frugiperda* (FAW) using a laboratory bioassay and assessments of larval density, leaf damage severity, and plant injury incidence in the field confirmed that transgenic maize LD05 could play an important role in FAW management in China and be an effective component for integrated FAW management in China.

## Figures and Tables

**Table 1:** Determination of the anti-insect activity and weight inhibition dose of FAW against M2CryAb-VIP3A protein.

Bt Protein	N	Regression Equation	LC_50_ (95%FL) (μg cm^−2^)	LC_95_ (95%FL) (μg cm^−2^)	GIC_50_ (95%FL) (μg cm^−2^)	GIC_95_ (95%FL) (μg cm^−2^)
M2CryAb-VIP3A	720	y = 1.246 + 2.012x	0.024 (0.001~0.062)	0.508 (0.217~14.296)	-	-
M2CryAb-VIP3A	720	y = 1.229 − 1.347x	-	-	0.142 (0.120~0.162)	0.556 (0.482~7.146)

**Table 2:** Comparative analysis of agronomic characters between LD05 and Zheng58.

Agronomic Trait	LD05 (2022)	Zheng58 (2022)	LD05 (2023)	Zheng58 (2023)	LD05 (2024)	Zheng58 (2024)
Tasseling stage (d)	52.4 ± 0.5 a	52.2 ± 0.4 a	52.3 ± 0.7 a	51.9 ± 0.6 a	52.3 ± 0.5 a	52.2 ± 0.8 a
Silking stage (d)	56.9 ± 0.6 a	56.9 ± 0.6 a	56.8 ± 0.4 a	57.1 ± 0.3 a	57.0 ± 0.8 a	56.7 ± 1.0 a
Pollen stage (d)	57.1 ± 0.3 a	57.3 ± 0.5 a	57.4 ± 0.5 a	57.3 ± 0.7 a	57.2 ± 0.4 a	57.3 ± 0.7 a
Growth stage (d)	109.0 ± 0.5 a	109.1 ± 0.6 a	109.2 ± 0.4 a	109.2 ± 0.8 a	109.3 ± 0.5 a	108.9 ± 0.6 a
Plant height (cm)	155.9 ± 1.4 a	155.7 ± 1.3 a	155.2 ± 1.1 a	155.5 ± 1.4 a	155.2 ± 1.1 a	155.9 ± 1.3 a
Ear height (cm)	54.4 ± 0.6 a	54.3 ± 0.9 a	54.3 ± 0.5 a	54.5 ± 0.9 a	54.4 ± 0.5 a	54.5 ± 0.6 a
Rows per ear	12.0 ± 0 a	12.0 ± 0 a	12.0 ± 0 a	12.2 ± 0 a	12.0 ± 0 a	12.0 ± 0 a
Ear diameter (cm)	3.84 ± 0.07 a	3.87 ± 0.5 a	3.82 ± 0.08 a	3.86 ± 0.06 a	3.85 ± 0.05 a	3.84 ± 0.06 a
100-kernel weight (g)	34.5 ± 0.3 a	34.3 ± 0.5 a	34.4 ± 0.5 a	34.6 ± 0.4 a	34.4 ± 0.5 a	34.5 ± 0.5 a
Pollen diameter (µm)	89.7 ± 2.6 a	89.9 ± 3.1 a	89.4 ± 3.7 a	89.8 ± 3.5 a	91.6 ± 2.8 a	90.8 ± 3.3 a

Data in the table represent means ± SD. Different letters denote significant differences (*p* < 0.05) between transgenic LD05 and its non-transgenic Zheng58 across three years (2022–2024) based on Tukey HSD test.

**Table 3:** Pollen germination rates of transgenic maize LD05 and non-transgenic Zheng58 under differential treatment conditions.

Temperature Treatment	Processing Time	Germination Rate of LD05 (%) (2022)	Germination Rate of Zheng58 (%) (2022)	Germination Rate of LD05 (%) (2023)	Germination Rate of Zheng58 (%) (2023)	Germination Rate of LD05 (%) (2024)	Germination Rate of Zheng58 (%) (2024)
—	0 min	74.22 ± 2.04 a	74.69 ± 2.07 a	76.43 ± 2.62 a	76.93 ± 1.73 a	75.73 ± 3.01 a	75.92 ± 1.77 a
25 °C	1 h	76.23 ± 2.61 a	75.45 ± 3.06 a	74.96 ± 2.22 a	74.40 ± 3.00 a	75.25 ± 3.18 a	75.93 ± 3.12 a
	3 h	30.65 ± 1.88 a	30.51 ± 1.63 a	31.92 ± 2.14 a	31.70 ± 2.90 a	29.70 ± 2.01 a	30.96 ± 2.74 a
	6 h	3.12 ± 0.28 a	2.84 ± 0.26 a	2.83 ± 0.22 a	2.98 ± 0.30 a	2.71 ± 0.33 a	3.18 ± 0.22 a
30 °C	1 h	75.58 ± 2.80 a	75.41 ± 3.82 a	74.81 ± 4.31 a	76.07 ± 3.96 a	75.71 ± 3.19 a	78.11 ± 2.56 a
	3 h	24.67 ± 3.75 a	26.10 ± 5.41 a	23.63 ± 4.63 a	26.53 ± 5.82 a	25.41 ± 5.29 a	23.28 ± 5.01 a
	6 h	0.00 ± 0.00 a	0.00 ± 0.00 a	0.00 ± 0.00 a	0.00 ± 0.00 a	0.00 ± 0.00 a	0.00 ± 0.00 a
35 °C	1 h	58.35 ± 4.07 a	59.53 ± 2.77 a	63.44 ± 5.03 a	60.91 ± 6.00 a	61.74 ± 5.17 a	57.35 ± 4.57 a
	3 h	19.08 ± 3.70 a	20.97 ± 4.92 a	19.53 ± 4.26 a	20.27 ± 3.70 a	18.65 ± 3.53 a	17.24 ± 4.01 a
	6 h	0.00 ± 0.00 a	0.00 ± 0.00 a	0.00 ± 0.00 a	0.00 ± 0.00 a	0.00 ± 0.00 a	0.00 ± 0.00 a

Data are presented as means ± SD. Different letters indicate statistically significant differences (*p* < 0.05) between transgenic LD05 and its non-transgenic Zheng58 across three consecutive years (2022–2024), as determined by Tukey HSD test.

## Data Availability

The data presented in this study are available in the graphs and tables provided in the manuscript.
